# Editorial: Sarcopenic Obesity: Mechanisms and Countermeasures

**DOI:** 10.3389/fnut.2022.886323

**Published:** 2022-03-23

**Authors:** Andrew J. Murton, Marlou L. Dirks, Benjamin T. Wall

**Affiliations:** ^1^Department of Surgery, University of Texas Medical Branch, Galveston, TX, United States; ^2^Sealy Center of Aging, University of Texas Medical Branch, Galveston, TX, United States; ^3^Nutritional Physiology Group, Department of Sport and Health Sciences, College of Life and Environmental Sciences, University of Exeter, Exeter, United Kingdom

**Keywords:** sarcopenic obesity, muscle metabolism, muscle protein turnover, dietary interventions, frailty

Sarcopenic obesity, the age-related loss of skeletal muscle mass that occurs concomitant with increased adiposity, has a dramatic effect on the functional ability and metabolic health of affected individuals. However, it is thought that in many cases sarcopenic obesity goes undetected for prolonged periods, with diagnosis occurring when functional and clinical manifestations are already evident. There is therefore a pressing need to understand the biological and metabolic etiology of sarcopenic obesity, coupled with a detailed understanding of the influence that social and economic factors have on its prevalence and progression. Addressing these knowledge gaps represents an essential precursor for the development of effective countermeasures that can prevent and/or treat the occurrence of sarcopenic obesity. In response, a special issue in *Frontiers Nutrition* on “Sarcopenic Obesity: Mechanisms and Countermeasures” was recently commissioned and included a collection of seven articles discussed below.

The association between the loss of muscle mass and increased adiposity through the lifespan has been observed by several groups, yet the precise characterization and/or diagnosis of sarcopenic obesity remains ambiguous, compromising the delivery of care to affected individuals. In the detailed review by Morgan et al. included in the special issue, the authors highlight the overall lack of consensus in the clinical and scientific communities as to suitable defining criteria, resulting in multiple competing definitions. Despite these challenges, the authors highlight the advantageous that imaging (e.g., MRI, DEXA) and direct measures of muscle strength and/or physical performance offer over traditional rudimentary anthropometric measures to identify sarcopenic obese individuals. However, as cautioned by Mendham et al., access to required specialist equipment and staff may be restricted or non-existent in underdeveloped and developing nations. Consequently, the incidence of sarcopenic obesity in developing nations is likely underappreciated, complicating the assignment of resources for treatment and preventative care in overstretched health care systems. In response, unique and innovative solutions are required that can be readily applied in resource restricted areas, allowing the identification of at-risk individuals and implementation of appropriate treatment strategies.

While the true global incidence of sarcopenic obesity remains unclear, recent advancements in the study of the interplay between protein and lipid metabolism is beginning to reveal key physiological and molecular regulatory mechanisms that may underpin development of the condition. One such phenomenon which has garnered increased interest in recent years is the association between obesity and the inability of muscle to mount an anabolic response to exogenous amino acids. In the special issue, El Ayadi et al. investigate whether obesity is directly responsible for the development of muscle's insensitivity to nutrition, utilizing an established rodent model of diet-induced obesity. They demonstrate that a calorie-dense diet that induces hyperphagia fails to impact muscle protein synthetic capacity in response to food, irrespective of age. Curiously, the authors observed that old rodents, unlike their young counterparts, fail to gain weight in response to increased energy intake, in part explained by the old animals' ability to increase whole-body energy expenditure in response to the experimental diet. Identification of the underlying mechanisms responsible for this effect could aid the development of novel treatments to support the aging obese human.

While gaps remain in our understanding of the physiological and biological events that underpin sarcopenic obesity, novel treatment approaches are starting to emerge. To date, most work has focused on the role that physical activity, exercise, and diet can play in the prevention and treatment of sarcopenic obesity. To this end, Zhang et al., undertaking a meta-analysis of published work, report that dietary omega-3 polyunsaturated fatty acids (PUFA) are inversely associated with sarcopenia, reinforcing the general consensus that omega-3 PUFA supplementation could be beneficial to the aging adult. Likewise, in their perspective article, Schourfour et al., highlight the virtues of a modest hypocaloric diet coupled with resistance exercise and protein supplementation as a strategy to counteract sarcopenic obesity. Though the authors caution that hypocaloric diets can lead to protein restriction and compromise the retention of muscle mass if not appropriately managed. This, coupled with the challenges of implementing exercise and restrictive dietary programs in sedentary and/or overweight individuals, has led to the development of novel digital technologies geared toward eliciting controlled long-term behavior change; although the authors note that issues with digital competency, visual impairment, and reduced fine motor skills could act as barriers to their wide-scale implementation in elderly populations.

While hypocaloric diets can risk protein malnutrition, two articles in the special issue offer strategies that could prove to protect muscle mass during times of calorie restriction. Macedo de Castro et al. observed that diets supplemented with nicotinamide riboside, a compound analogous to vitamin B3, decrease body weight in rats without impacting gastrocnemius muscle weight. Given the observed increase in muscle weight relative to body weight, the approach could prove beneficial for the return of functional independence in treated individuals. Notably, the authors observed that weight loss occurred without compromising muscle mass irrespective of whether animals were fed a calorie restricted, standard, of calorie dense diet, suggesting that the approach could be coupled to treatments involving hypocaloric diets, or applied in individuals where dietary compliance remains challenging. Alternatively, de Marco Castro et al. suggested that the gut microbiota could represent a promising target to enhance the anabolic response seen in muscle to a given quantity of protein. They contend that the gut microbiota could be manipulated to aid the digestibility of protein containing foods, increasing the resultant aminoacidemia and thereby enhance the synthesis of muscle contractile proteins. As highlighted by the authors, while data from murine models supports a link between the gut microbiota and muscle metabolic function, whether manipulating the gut microbiota influences human muscle protein anabolism remains to be determined.

In summary, the goal of the special issue was to provide a contemporary overview of the scientific research concerning the development and treatment of sarcopenic obesity. The special issue highlights the great strides have been made in our understanding of the condition, but reinforces the need for research across the translational continuum to develop and implement nutritional, physical activity and pharmacological countermeasures that can support healthy aging.

## Author Contributions

All authors listed have made a substantial, direct, and intellectual contribution to the work and approved it for publication.

## Funding

AJM is supported in part by a grant from the National Institute of Aging (P30-AG024832).

## Conflict of Interest

The authors declare that the research was conducted in the absence of any commercial or financial relationships that could be construed as a potential conflict of interest.

## Publisher's Note

All claims expressed in this article are solely those of the authors and do not necessarily represent those of their affiliated organizations, or those of the publisher, the editors and the reviewers. Any product that may be evaluated in this article, or claim that may be made by its manufacturer, is not guaranteed or endorsed by the publisher.

